# Predicting Cetacean Habitats from Their Energetic Needs and the Distribution of Their Prey in Two Contrasted Tropical Regions

**DOI:** 10.1371/journal.pone.0105958

**Published:** 2014-08-27

**Authors:** Charlotte Lambert, Laura Mannocci, Patrick Lehodey, Vincent Ridoux

**Affiliations:** 1 Centre d'Études Biologiques de Chizé-La Rochelle, UMR 7372 Université de La Rochelle - CNRS, Institut du Littoral et de l'Environnement, La Rochelle, France; 2 Marine Geospatial Ecology Lab, Duke University West Campus, Durham, North Carolina, United States of America; 3 MEMMS (Marine Ecosystems Modeling and Monitoring by Satellites), CLS, Space Oceanography Division, Ramonville, France; 4 Observatoire PELAGIS, UMS 3462 Université de La Rochelle - CNRS, Systèmes d'Observation pour la Conservation des Mammifères et des Oiseaux Marins, La Rochelle, France; Aristotle University of Thessaloniki, Greece

## Abstract

To date, most habitat models of cetaceans have relied on static and oceanographic covariates, and very few have related cetaceans directly to the distribution of their prey, as a result of the limited availability of prey data. By simulating the distribution of six functional micronekton groups between the surface and ≃1,000 m deep, the SEAPODYM model provides valuable insights into prey distributions. We used SEAPODYM outputs to investigate the habitat of three cetacean guilds with increasing energy requirements: sperm and beaked whales, *Globicephalinae* and *Delphininae*. We expected High Energy Requirements cetaceans to preferentially forage in habitats of high prey biomass and/or production, where they might easily meet their high energetic needs, and Low Energy Requirements cetaceans to forage in habitats of either high or low prey biomass and/or production. Cetacean sightings were collected from dedicated aerial surveys in the South West Indian Ocean (SWIO) and French Polynesia (FP). We examined cetacean densities in relation to simulated distributions of their potential prey using Generalised Additive Models and predicted their habitats in both regions. Results supported their known diving abilities, with *Delphininae* mostly related to prey present in the upper layers of the water column, and *Globicephalinae* and sperm and beaked whales also related to prey present in deeper layers. Explained deviances ranged from 9% for sperm and beaked whales in the SWIO to 47% for *Globicephalinae* in FP. *Delphininae* and *Globicephalinae* appeared to select areas where high prey biomass and/or production were available at shallow depths. In contrast, sperm and beaked whales showed less clear habitat selection. Using simulated prey distributions as predictors in cetacean habitat models is crucial to understand their strategies of habitat selection in the three dimensions of the ocean.

## Introduction

Since many marine top predators, especially cetaceans, are currently in decline [1], establishing models that correctly describe and predict their preferred habitats is critical to develop appropriate conservation strategies. However, pelagic ecosystems are vast dynamic and complex systems that represent specific challenges for modelling cetacean habitats. Oceanographic processes are characterised by spatial or temporal lags between physical features and the resulting biological responses [2]. To provide robust indicators of cetacean distributions, environmental predictors of habitats must be carefully selected and be representative of the ecological processes underlying these distributions.

To date, cetacean habitats have mostly been modelled using oceanographic features and primary production as predictors [3]–[6]. Cetaceans were shown to be related to bottom depth or slope, sea surface temperature, nutrient concentrations, as well as primary production. Such predictors are assumed to be good indicators of the distribution of lower trophic levels and subsequently of the entire food web. However, the main issue with such distal predictors is the existence of potential lags between oceanographic processes and biological response at upper trophic levels. To ensure that models explore direct biological relationships and avoid these lags, habitat modelling should be performed with predictors as close as possible to driving variables [7], namely prey distribution, since cetaceans are very sensitive to variations in prey abundance or quality [8]. However, data on prey distributions are not available at large spatiotemporal scales, explaining the scarcity of studies incorporating prey abundance in habitat models.

Another advantage of incorporating prey distribution in habitat models is that it provides a good tool to explore foraging strategies of cetaceans at large scale. Spitz and colleagues [9] demonstrated that energetic requirements governed cetacean foraging strategies, in accordance with the Optimal Foraging Theory [10]. Extrapolating this strategy of prey selection to habitats, Mannocci and colleagues [11], [12] found that the general energetic requirements of cetaceans governed their strategies of habitat selection over large spatial scales. High Energy Requirements (HER) cetaceans preferentially exploited habitats of high primary production, whereas Low Energy Requirements (LER) cetaceans were less sensitive to habitat quality, as expressed by lower primary production. These results were obtained using habitat models based on cetacean sightings collected from aerial surveys in the South West Indian Ocean (SWIO) [11] and French Polynesia (FP) [12], together with distal static and oceanographic predictors.

In the present study, we aimed to further explore this hypothesis, using prey distributions as proximal predictors of habitat quality. In the absence of synoptic datasets of prey, *i.e.* mainly micronekton, at such large spatial scales, we turned to numerical models. One original modelling approach has been developed [13] as a component of the Spatial Ecosystem And Population Dynamics Model (SEAPODYM). This model describes six functional groups of prey defined by their daily vertical migration patterns in three biological layers from *c.* 0 to ≃1,000 m. It has been successfully used to analyse tuna catch data [14], simulate habitats and movements of turtles [15] and predict the large-scale population dynamics of several tuna species based on hindcast simulations from coupled physical-biogeochemical models [13], [16]–[18].

In this study, we developed habitat models of cetaceans based on the same sightings as Mannocci and colleagues [11] in the SWIO and Mannocci and colleagues [12] in FP for the same three energetic guilds of cetaceans (sperm and beaked whales, *Globicephalinae* and *Delphininae*). Instead of predicting habitat based on static and oceanographic variables, we implemented Generalised Additive Models to highlight relationships between cetacean densities and simulated distributions of their potential prey. We predicted cetacean habitats at the regional scale and highlighted the differences between the SWIO and FP. Since SEAPODYM outputs provide biomass and productivity of potential prey of cetaceans and match the survey periods, their use as predictors should limit spatial and temporal lags with the response variable.

**Figure 1:**
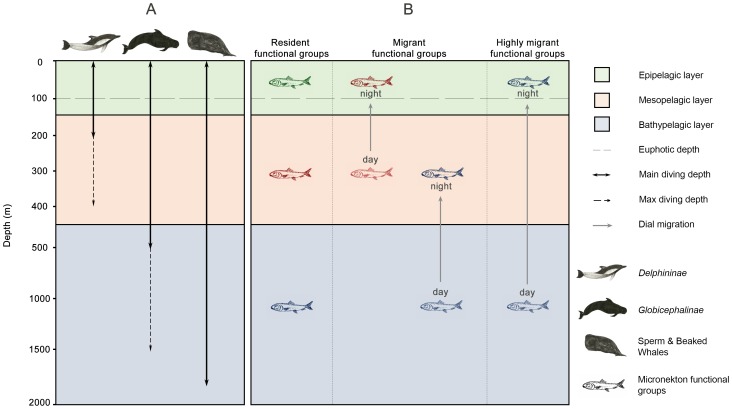
Vertical repartition of cetaceans and prey in the water column. The three layers are defined as in SEAPODYM, for an average situation with an euphotic depth of 100 m. A: Average and maximum diving depths of the three studied cetacean guilds (*Delphininae*, *Globicephalinae* and sperm and beaked whales). B: the six micronekton functional groups as defined in SEAPODYM, classified according to their daytime layer and their daily vertical migration (see text for details).

As Mannocci and colleagues [11], [12], we wanted to explore to what extent the energy requirements of cetaceans govern their strategies of habitat selection. Therefore, we expected HER cetaceans to have narrower foraging habitats, focusing on areas with high prey availability to sustain their high energy requirements, whereas LER cetaceans would use a wider range of prey availability. Moreover, since SEAPODYM outputs provide a third dimension (depth), we investigated emerging statistical relationships between prey groups of different water layers and cetaceans diving abilities (Figure 1A). *Delphininae* would be able to access mostly prey inhabiting epipelagic and mesopelagic layers, whereas *Globicephalinae* and sperm and beaked whales would be able to exploit prey up to the bathypelagic layer.

## Data and Methods

### Aerial Surveys

Two tropical regions were studied: the SWIO and FP, which allowed cetacean habitats to be studied in a large range of environmental conditions. The SWIO can be divided into three ecoregions: the Mozambique Channel, the Seychelles and the Mascarene Islands area. The Mozambique Channel is characterised by a very dynamic system of mesoscale eddies [19], [20], inducing a pronounced enhancement of phytoplankton production [21]. Enhanced productivity is also associated with mid-ocean shallow banks of the Seychelles plateau [21], whereas the Mascarenes are characterised by low-nutrient subtropical waters [22].

French Polynesia extends from the southern border of the productive equatorial upwelling (cold tongue) to the core of the South Pacific oligotrophic gyre, where downwelling precludes any upward flow of nutrients [23], defining a clear latitudinal productivity gradient. In the north, the Marquesas Islands are associated with a significant enhancement of phytoplankton production owing to an island mass effect [24] and the proximity of the equatorial upwelling. In the south, extreme nutrient scarcity in the euphotic layer results in a very low primary production [25].

**Figure 2:**
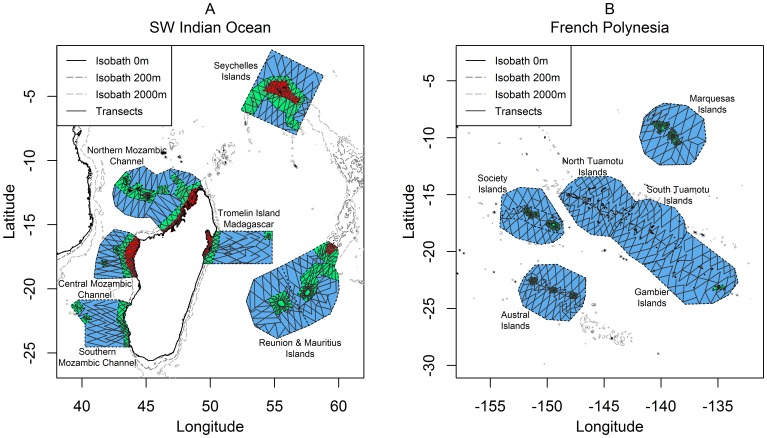
Study regions: South West Indian Ocean (A) and French Polynesia (B). Six geographic sectors were sampled in each region. Each sector was subdivided into bathymetric strata: neritic strata (red; absent in French Polynesia), slope strata (green) and oceanic strata (blue). Sampled transect lines are represented by solid lines inside sectors. For details on the sampling design, see [11], [12].

Aerial surveys were conducted during the austral summer in the SWIO (December 2009–April 2010) and FP (January–May 2011) (Figure 2). In the SWIO, the sampled region encompassed an area from 1 to 27°S and from 39 to 61°E. Six geographic sectors were sampled to encompass the three ecoregions: Northern, Central and Southern Mozambique Channel, The Seychelles, Tromelin-Madagascar and Reunion-Mauritius. French Polynesia lies between 5 and 30°S, and 156 and 132°W and includes five archipelagos, corresponding to the following sampled sectors covering the entire latitudinal productivity gradient: Marquesas, Society, North and South Tuamotu, Gambier and Australs Archipelagos.

Data were collected following the same standardised aerial protocol in both regions. This was based on a line-transect methodology [26], in which the angle to the track line was recorded for each observation. This angle, together with the flight altitude, informed about the perpendicular distance from the track line, required for the estimation of effort per grid cell (estimation of the effective strip width by distance sampling, ESW see [11] and [12]). Survey platforms were high-wing double engine aircrafts equipped with bubble windows, allowing a vertical observation of the sea surface. Sightings and group size were recorded at the lowest possible taxonomic level. Beaufort sea-state, turbidity, glare severity, cloud coverage and an index of subjective conditions were also collected on board as detection covariates.

The total survey effort was 83,726 km in the SWIO [11] and 98,476 km in FP [12]. Observed encounter rates collected on effort amounted 0.0282 *Delphininae* per km^2^ in SWIO and 0.0018 in FP, 0.176 *Globicephalinae* per km^2^ in SWIO and 0.022 in FP and 0.007 individuals per km^2^ in SWIO against 0.004 in FP for sperm and beaked whales (see Figure S1 and Table S1, and Mannocci and colleagues [11], [12] for details on the sightings). Data can be found online on the PELAGIS Observatory website (http://www.observatoire-pelagis.cnrs.fr).

### Cetacean guilds

The vast majority of cetaceans sighted during the survey (conducted in the austral summer) were odontocetes. The classification of odontocetes species into guilds relied on their energetic costs. Spitz and colleagues [9] demonstrated that HER species have a high muscular mitochondrial density and lipid content because their metabolism burns O_2_ at high rates, whereas LER species have a lower muscular mitochondrial density and lipid content due to lower O_2_ consumption. Since diving abilities are restricted by energetic capacities, the energetic costs of living of cetaceans mirror their diving abilities (depth and duration): species that have high capacities to save oxygen and reduced energy requirements are able to perform deep and long dives. Therefore, we used diving abilities as a proxy for their energy requirements to classify cetacean species into three guilds: sperm and beaked whales, *Globicephalinae and Delphininae* (from deeper to shallower divers, see [11] and [12] for the species composition of these guilds).

Prey types foraged on by studied cetacean species roughly match the size range of micronekton as defined in SEAPODYM (prey of 1 to 20 cm in size). All cetacean species studied here forage on prey included in this size range, although for most of them, the upper limit of the observed range size of consumed prey was greater than the upper limit of the modelled range. Concerning *Delphininae*, the mean prey sizes rarely exceed 20 cm [27]–[32]. For *Globicephalinae*, the range of prey size also encompasses the SEAPODYM size window [27], [33], [34]. Lastly, size range of prey consumed by sperm and beaked whales also overlap the SEAPODYM size window [27], [34]–[36]. Although sperm whales are known to prey upon giant squids, Evans & Hindell [36] showed that squids greater than 1 m in size represented only 0.6% of all the cephalopods present in diet samples, and individuals smaller than 30 cm represented 73.5% of the diet.

### SEAPODYM outputs

The SEAPODYM model simulates several functional groups of micronekton [13], used initially to predict the distribution of tuna populations [16]. In the present study, only predicted micronekton distributions were used. Micronekton encompasses actively swimming organisms roughly in the size range of 1–20 cm, including cephalopods, crustaceans, fishes and jellyfishes [37].

Micronekton modelling is driven by temperature, currents, primary production and euphotic depth. The physical variables are provided by the Mercator-Ocean GLORYS-2v1 (GLobal Ocean ReanalYsis and Simulations) reanalysis. Net primary production and associated euphotic depth are derived from ocean colour satellite data (http://www.science.oregonstate.edu/ocean.productivity/), using the Vertically Generalised Production Model (VGPM) [38]. GLORYS 2v1 is an eddy-permitting global ocean reanalysis produced for the 1992–2009 period, with the ocean general circulation model configuration ORCA025 NEMO [39] at a resolution of 0.25°×1 day and an assimilation method adapted to this configuration [40]. Because satellite (sea surface temperature and altimetry) and *in situ* data are assimilated into this ocean reanalysis, predicted fields of temperature and currents are coherent with those of primary production derived from ocean colour data. Both GLORYS-2v1 outputs and primary production data were interpolated onto a regular 0.25°×0.25° grid with a weekly time step to be used as forcing for the SEAPODYM model.

In SEAPODYM, the functional groups of micronekton are characterised by their time of development relative to water temperature [13], through allometric relationships. Each functional group is modelled as a single multi-species population, with continuous mortality rates and recruitment. Organisms are recruited into the micronekton population when they reach a minimum weight of 1 g (also estimated on the basis of the time of development related to water temperature) and disappear from it when they either die or exceed the maximum size. Production is thus defined as the amount of energy transferred at the time of recruitment in the functional group.

Micronekton is characterised by nycthemeral vertical migrations induced by daylight variations. Such behaviour is thought to be mainly a predator avoidance strategy: micronekton sinks to deeper layers during daytime where predation pressure is lower [41]. The SEAPODYM model includes six functional groups defined according to their migration patterns (Figure 1B): epipelagic, non-migrant mesopelagic, migrant mesopelagic, non-migrant bathypelagic, migrant bathypelagic and highly migrant bathypelagic [13]. Migrant mesopelagic and highly migrant bathypelagic organisms spend the night in the epipelagic layer and move back to their respective layers during the day. Migrant bathypelagic organisms also perform a migration, but only between mesopelagic and bathypelagic layers.

The three layers are defined relative to the euphotic depth (Figure S2). In the most recent version of SEAPODYM, the boundary between epipelagic and mesopelagic layers is defined as 1.5 euphotic depths, whereas the limit between mesopelagic and bathypelagic layers is 4.5 euphotic depths. The SEAPODYM prey population model has been parameterised and evaluated with biomass estimates from micronekton sampling cruises (*e.g.*, [42], [43]) and ADCP backscatter data [18].

### Data processing

Effective strip widths (ESWs) were estimated for each cetacean guild by fitting detection functions to perpendicular distances using multiple covariate distance sampling [44] to model the effect of both distance and detection conditions on detection probability. Results are presented in [11] for the SWIO and [12] for FP. To model cetacean habitats, we used micronekton outputs from SEAPODYM as predictors. Euphotic depth (as used in SEAPODYM and computed from the VGPM model) was included, to consider variations in vertical accessibility of micronekton to cetaceans between the different geographic sectors. Since SEAPODYM outputs were provided at a 0.25°×0.25° spatial resolution, cetacean sightings and sampled surface areas were aggregated at the same temporal resolution on the same horizontal grid using ArcGIS 10 [45]. The observed numbers of individuals as well as sampled surface areas were summed in each grid cell. The sampled surface area for each single transect was the transect length multiplied by twice the corresponding associated ESW.

Both production and biomass of each micronekton functional group (*i.e.*, 12 variables) were tested as potential predictors. As noted above, the production represents the new cohort of organisms recruited into the micronekton group when they reach the minimum weight fixed at 1 g. Therefore, it can provide different information on the behaviour of predators. The selection of production rather than biomass for a given group would suggest that predators have a preference for targeting prey of small sizes, but that are easier to catch and in greater abundance than the older and larger organisms accumulated into the biomass of the corresponding micronekton group. The spatial distributions of these functional groups are described in Text S1 and Figures S3 and S4.

### Habitat modelling

We used Generalised Additive Models (GAMs; [46]) to model the relative densities of the three cetacean guilds. We used a quasi-Poisson distribution with variance proportional to the mean, because the dispersion of our data was greater than that predicted by the classical Poisson distribution (over-dispersion; [47], [48]). The mean of the response variable was related to the additive predictor by a log-link function.

The relationship between the response variable (number of individuals per pixel *i*) and the additive predictor was modelled in both regions as 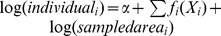
where 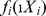 is a non-parametric smooth function (spline) of the covariate *X*, and 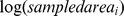 is the model offset. This offset allowed the variation in the amount of effort per pixel to be taken into account [49]. Micronekton biomass and production were log-transformed prior to model selection to limit outliers. To avoid over-fitting of the data while allowing the curve to be non-linear, we constrained the maximum number of degrees of freedom for each spline to three.

A selection procedure was implemented based on models with between one and four covariates, excluding all combinations of covariates with a pair-wise correlation higher than 0.7 in the SWIO, and 0.8 in FP (Table S2). These correlations were calculated with the Spearman correlation test implemented in R [50], using the Hmisc package [51]. The selection criterion was the Generalised Cross-Validation score (GCV, the lower the better). The GCV score is a prediction error criterion: it estimates the mean error, by removing each datum in turn and re-predicting it from the model fitted to the remaining data [52]. For each selected model, we quantified the contribution of each covariate to the linear predictor, as a percentage [53].

Finally, we produced prediction maps for each guild for both regions based on the selected models using SEAPODYM outputs averaged over the period of each survey (3–4 months). We predicted only within the range of sampled covariate values (model-based interpolation; [54]) to avoid extrapolation. In order to allow comparisons between cetacean guilds and regions, we provided relative density maps, showing predicted densities (individuals per km^2^) normalized by the highest predicted density over the two regions and the three guilds (here, *Globicephalinae* in SWIO). Moreover, we mapped uncertainty of the model parameters with the coefficient of variation (CV). Model selections, the quantification of contributions and predictions were performed in R using the mgcv package, especially gam and predict.gam functions [52], [53].

## Results

### Cetacean habitat models

#### *Delphininae*

**Figure 3:**
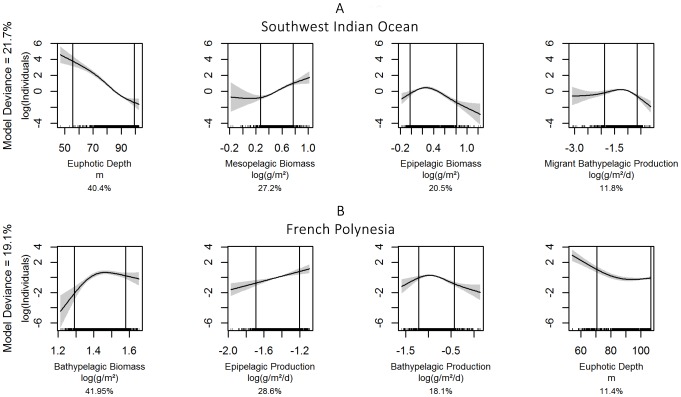
Smoothed functions for the selected covariates for *Delphininae* in the two study regions. The South West Indian Ocean model is shown in panel A, French Polynesia model in panel B. The solid line is the smooth function, shaded regions represent the 95% confidence intervals. The percentage indicated below each graph is the contribution of each covariate in the linear predictor.

In the SWIO, the distribution of *Delphininae* was best predicted by a model containing, in order of decreasing contributions: euphotic depth, non-migrant mesopelagic biomass, epipelagic biomass and migrant bathypelagic production (Table 1). This model explained 21.7% of the deviance. In the 5–95% quantile interval (corresponding to the core data; Figure 3A), relationships were negative with euphotic depth, positive with non-migrant mesopelagic biomass and unimodal with the other two covariates.

**Table 1:** Selected models for the three cetacean guilds in the SWIO and FP.

			*Delphininae*		*Globicephalinae*		Sperm and Beaked Whales	
			South West Indian Ocean	French Polynesia	South West Indian Ocean	French Polynesia	South West Indian Ocean	French Polynesia
Micronekton functionnal groups	Biomass	Epipelagic	20.5	-	-	-	-	-
		Mesopelagic	27.2	-	20.8	11.4	-	-
		Migrant Mesopelagic	-	-	-	-	-	37.5
		Bathypelagic	-	41.95	-	60.3	-	-
		Migrant Bathypelagic	-	-	-	-	-	23.9
		Highly Migrant Bathypelagic	-	-	-	-	-	-
	Production	Epipelagic	-	28.6	29.7	-	15.6	-
		Mesopelagic	-	-	-	18.5	-	-
		Migrant Mesopelagic	-	-	-	-	-	-
		Bathypelagic	-	18.1	-	-	-	-
		Migrant Bathypelagic	11.8	-	-	9.8	23.5	20.8
		Highly Migrant Bathypelagic	-	-	21.8	-	36.3	-
Euphotic Depth			40.4	11.4	27.8		24.6	17.8
***Explained deviances (%)***			***21.7***	***19.1***	***17.7***	***47.1***	***8.7***	***12.2***

Contributions to linear predictor of each selected covariates per models are indicated in corresponding cases (in %). Blank cases indicate covariate has been tested, but not selected in the best model. Explained deviances (in %) are indicated below each model.

In FP, the selected model (19.1% of the explained deviance) also contained euphotic depth, but as the least contributory covariate (Table 1). The other selected covariates were non-migrant bathypelagic biomass and production and epipelagic production. In the 5–95% quantile interval (Figure 3B), relationships were positive with non-migrant bathypelagic biomass and epipelagic production, but negative with non-migrant bathypelagic production and euphotic depth.

#### *Globicephalinae*

**Figure 4:**
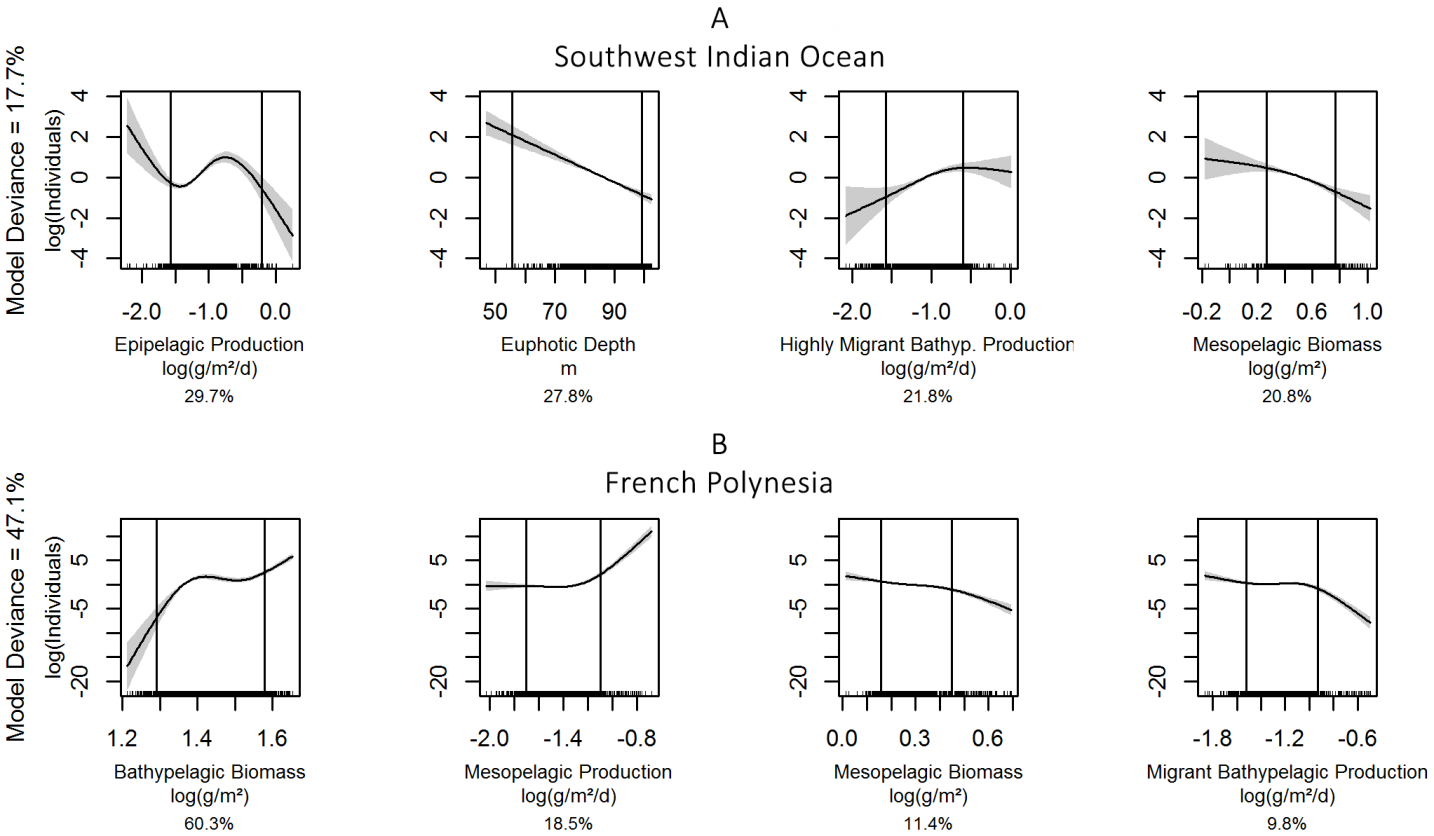
Smoothed functions for the selected covariates for *Globicephalinae* in the two study regions. The South West Indian Ocean model is shown in panel A, French Polynesia model in panel B. The solid line is the smooth function, shaded regions represent the 95% confidence intervals. The percentage indicated below each graph is the contribution of each covariate in the linear predictor.

In the SWIO, the selected model for *Globicephalinae* explained 17.7% of the deviance and contained, in order of decreasing contributions (Table 1): epipelagic production, euphotic depth, highly migrant bathypelagic production and non-migrant mesopelagic biomass. In the 5–95% quantile interval (Figure 4A), relationships were negative (and linear) for euphotic depth and non-migrant mesopelagic biomass, positive for highly migrant bathypelagic production and unimodal for epipelagic production.

In FP, euphotic depth did not appear among the predictors, with a model explaining 47.1% of the deviance (Table 1). The selected model contained non-migrant bathypelagic biomass, non-migrant mesopelagic biomass and production and migrant bathypelagic production. In the 5–95% quantile interval (Figure 4B), the relationship was positive with non-migrant bathypelagic biomass, whereas for the other three covariates the predicted density remained very close to zero, indicating a weak effect of these covariates.

#### Sperm and beaked whales

**Figure 5:**
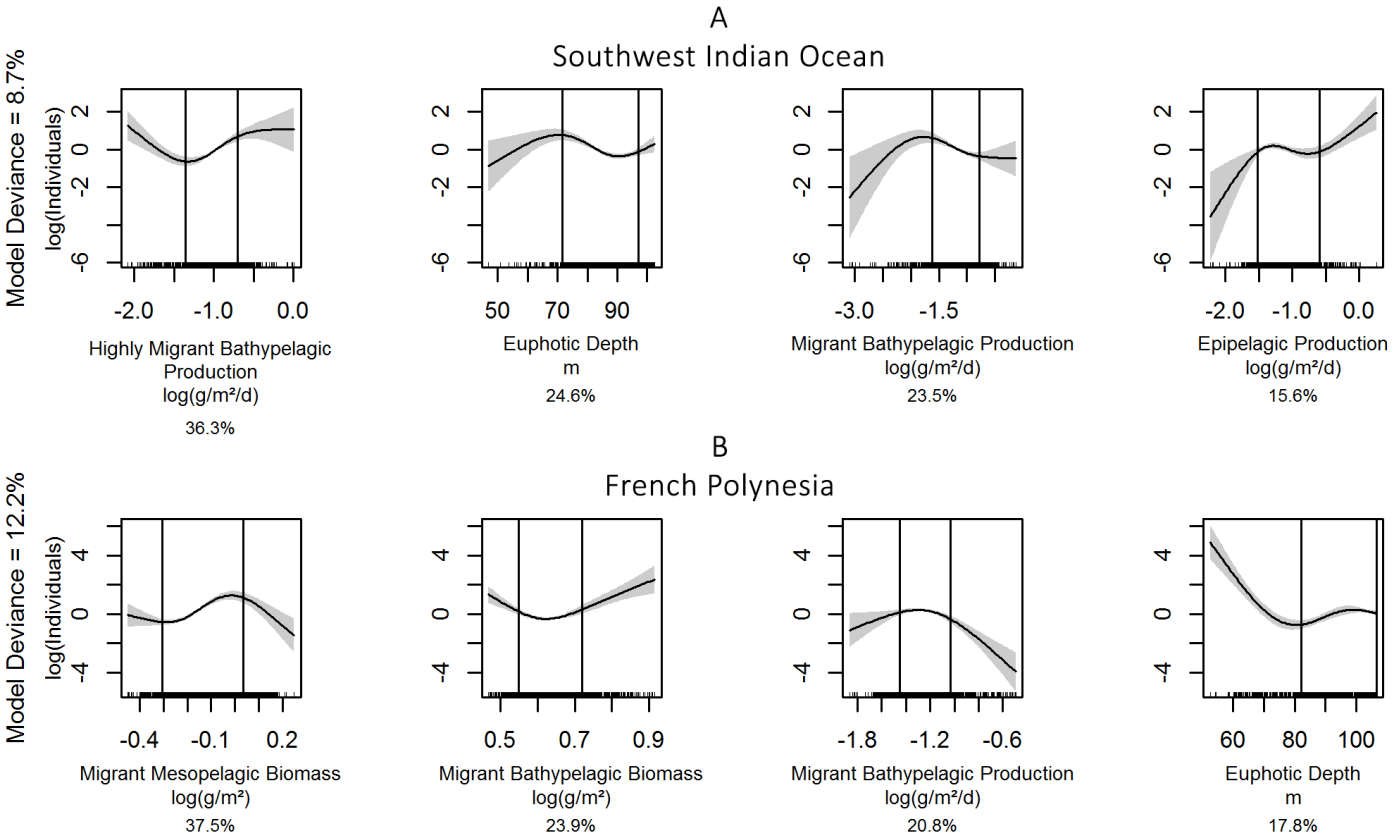
Smoothed functions for the selected covariates for sperm and beaked whales in the two study regions. The South West Indian Ocean model is shown in panel A, French Polynesia model in panel B. The solid line is the smooth function, shaded regions represent the 95% confidence intervals. The percentage indicated below each graph is the contribution of each covariate in the linear predictor.

The model for sperm and beaked whales had the lowest explained deviances, both in the SWIO and in FP, with 8.7% and 12.2%, respectively (Table 1). In the SWIO, their distribution was best predicted by highly migrant bathypelagic production, euphotic depth, migrant bathypelagic production and epipelagic production, in order of decreasing contributions. Relationships were negative for euphotic depth, migrant bathypelagic production and epipelagic production in the 5–95% quantile intervals (Figure 5A) but positive with highly migrant bathypelagic production.

In FP, the selected model included, in order of decreasing contributions (Table 1), migrant mesopelagic biomass, migrant bathypelagic biomass and production and euphotic depth. Relationships were complex in the 5–95% quantile intervals (Figure 5B), with unimodal relationships for the last three covariates, but a positive relationship with migrant mesopelagic biomass.

### Predicted distributions

#### *Delphininae*

**Figure 6:**
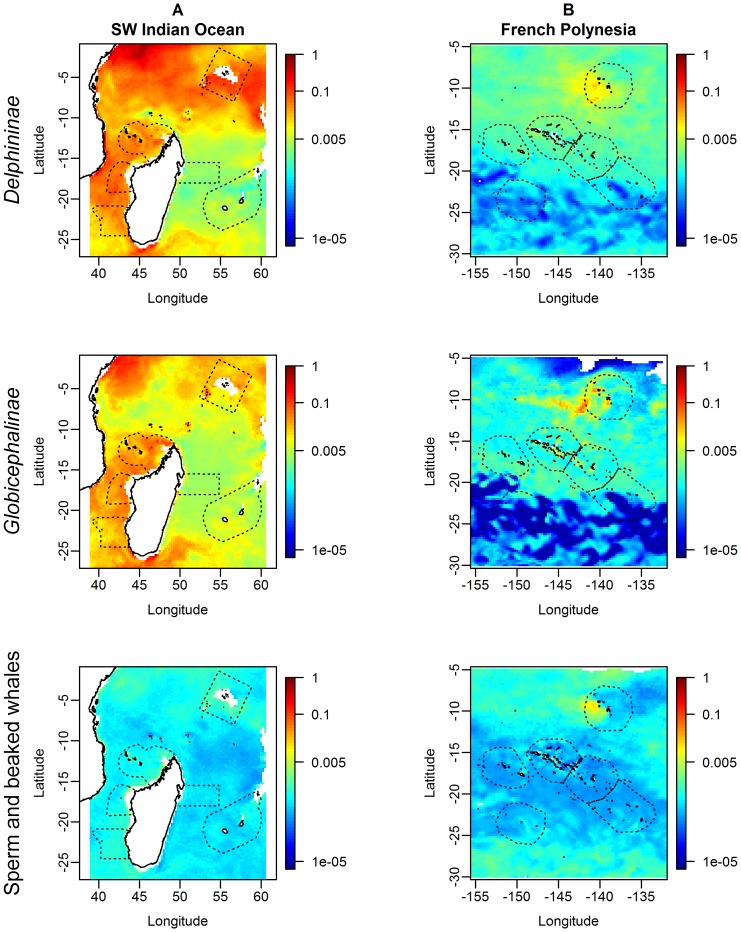
Predicted distributions (relative densities) for the three cetacean guilds in the two study regions. In the South West Indian Ocean (A) empty pixels were due to the absence of SEAPODYM outputs for the bathypelagic micronekton functional groups in the neritic strata (bathymetry <200 m). The limitation of predictions inside the range of sampled covariates values resulted in empty pixels in French Polynesia (B). In order to enhance contrast, we used a log colour scale.

**Figure 7:**
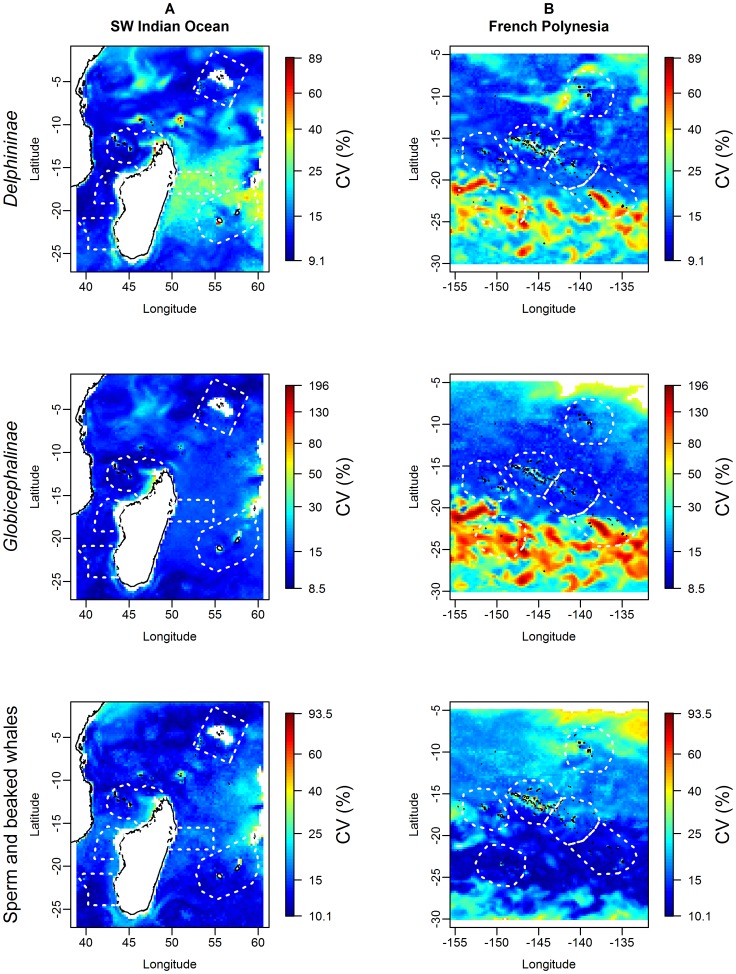
Uncertainty maps associated with the predictions for the three cetacean guilds in the two study regions. In the South West Indian Ocean (A) empty pixels were due to the absence of SEAPODYM outputs for the bathypelagic micronekton functional groups in the neritic strata (bathymetry <200 m). The limitation of predictions inside the range of sampled covariates values resulted in empty pixels in French Polynesia (B). In order to enhance contrast, we used the same colour scale (in log) for each guild in both regions but the colour scale is not comparable between the different guilds.

In the SWIO, high relative densities of *Delphininae* were predicted in the north of the region (especially off Kenya and southeast of the Seychelles), in the Mozambique Channel and south of Madagascar (Figure 6A). Lowest predicted densities were around the Mascarenes (Tromelin-Madagascar and Reunion-Mauritius). Mean predicted relative density was 3.8.10^−02^ over the whole region (Table 2), with a ratio of 17.8 between the highest and the lowest density sectors. Uncertainty was the lowest where density was the highest (Figure 7A).

**Table 2:** Statistics of the predictions for *Delphininae*, *Globicephalinae* and sperm and beaked whales.

	South West Indian Ocean			French Polynesia		
	Geographic sectors	Mean Predicted Relative Densities	Mean CV	Geographic sectors	Mean Predicted Relative Densities	Mean CV
*Delphininae*	NMC	2.6.10^−02^	14.80	MAR	3.8.10^−03^	17.90
	CMC	5.4.10^−02^	12.02	NTU	1.4.10^−03^	15.90
	SMC	2.5.10^−02^	14.20	STU	1.0.10^−03^	15.20
	RM	3.8.10^−03^	22.20	GAM	5.6.10^−04^	24.60
	SE	4.4.10^−02^	12.70	SOC	9.1.10^−04^	14.60
	TM	3.0.10^−03^	27.30	AUS	2.7.10^−04^	31.40
	*Whole Region*	*3.8.10^−02^*	*17.80*	*Whole Region*	*1.3.10^−03^*	*20.40*
	Between-sector ratio	17.79		Between-sector ratio	14.12	
*Globicephalinae*	NMC	2.7.10^−02^	14.70	MAR	5.9.10^−03^	18.10
	CMC	3.1.10^−02^	13.46	NTU	1.0.10^−03^	15.52
	SMC	2.9.10^−02^	13.13	STU	9.1.10^−04^	14.12
	RM	5.6.10^−03^	15.54	GAM	5.9.10^−04^	48.10
	SE	1.7.10^−02^	12.63	SOC	8.3.10^−04^	16.30
	TM	4.0.10^−03^	15.63	AUS	1.6.10^−04^	71.84
	*Whole Region*	*1.8.10^−02^*	*14.01*	*Whole Region*	*9.6.10^−04^*	*34.60*
	Between-sector ratio	7.58		Between-sector ratio	36.25	
Sperm and Beaked Whales	NMC	5.5.10^−04^	15.84	MAR	1.3.10^−03^	22.20
	CMC	6.1.10^−04^	16.48	NTU	2.2.10^−04^	19.05
	SMC	7.5.10^−04^	12.99	STU	2.0.10^−04^	16.60
	RM	3.9.10^−04^	14.82	GAM	2.2.10^−04^	12.40
	SE	7.5.10^−04^	13.90	SOC	2.6.10^−04^	17.76
	TM	3.7.10^−04^	16.30	AUS	4.2.10^−04^	11.90
	*Whole Region*	*5.3*.10^−04^	*14.90*	*Whole Region*	*5.4*.10^−04^	*18.40*
	Between-sector ratio	2.05		Between-sector ratio	6.14	

Mean relative predicted densities and associated mean uncertainty (CV, %) are given for each geographic sector and for the whole regions for the three guilds. The ratio between the highest and the lowest density sector is indicated for each region (between-sector ratio). In the South West Indian Ocean, NMC: Northern Mozambique Channel, CMC: Central Mozambique Channel, SMC: Southern Mozambique Channel, RM: Réunion-Mauritius, SE: Seychelles, TM: Tromelin-Madagascar. In French Polynesia, MAR: Marquesas, NTU: North Tuamotu, STU: South Tuamotu, GAM: Gambier, SOC: Society, AUS: Australs.

In FP, the model predicted much lower relative densities, with a regional mean of only 1.3.10-^03^ (Table 2). The highest relative densities were predicted in the southwest of the Marquesas and the lowest in the south of the region (Figure 6B), with a ratio of 14.1 between the highest and the lowest density sectors. Uncertainty was the lowest where density was the highest (the same range as in the SWIO, Figure 7B).

#### *Globicephalinae*

In the SWIO, the predicted distribution of *Globicephalinae* was similar to that of *Delphininae*: the highest relative densities were predicted off Kenya, south of Madagascar (close to the shelf) and in the Mozambique Channel, the lowest were predicted east of Madagascar and east of the Mascarene (Figure 6A). The mean predicted relative density was 1.8.10^−02^ over the region (Table 2). Uncertainty was low over the whole region, except close to Madagascar and the Mascarene shelves or slopes (Figure 7A). The between-sector ratio was 7.6.

In FP, the mean predicted relative density over the region was low (3.6.10^−04^, Table 2). The predictions were relatively homogeneous (Figure 6B), especially in the north. The highest relative densities were predicted west of the Marquesas, and the lowest, in the south of the region. A low uncertainty was associated with high densities, but it reached high values in the south, where predicted densities were the lowest (Figure 7B). The between-sector ratio was 36.3, the highest value of the three guilds.

#### Sperm and beaked whales

The sperm and beaked whale predicted distribution in the SWIO differed from that of the other two guilds, with a much lower mean relative density (5.3.10^−04^; Table 2). The highest relative densities were predicted close to the slopes of Madagascar (Mozambique Channel and south of the island), the Seychelles and off Kenya. The predicted distribution was more homogeneous than for *Delphininae* and *Globicephalinae*, with intermediate relative densities both in oligotrophic and productive waters (Figure 6A). The between-sector ratio was low compared to that of the other two guilds, with a ratio of only 2.1 between the highest and the lowest density sectors. An overall low uncertainty was associated with both high and low predicted densities (Figure 7A).

In FP, predicted relative densities were the lowest for sperm and beaked whales, with a mean of 5.4.10^−04^ (Figure 6B, Table 2). Sperm and beaked whales were homogenously distributed over FP, with the highest predicted relative densities in the north and south, and the lowest in central FP. Given the high relative densities predicted in the Marquesas compared to the other sectors, the between-sector ratio was 6.1. Uncertainties showed a different pattern compared to the other two guilds: lowest uncertainties were associated with low and intermediate predicted densities, whereas higher predicted densities were associated with higher uncertainties (Figure 7B). An area of high uncertainty was found in the northeastern FP.

A comparison of predictions from models with the distributions of sightings (Figure S1) shows a good agreement for all the three guilds in both regions.

## Discussion

### Costs of living and habitat selection

#### HER cetaceans(*Delphininae* and *Globicephalinae*)

*Delphininae* have the most energetically costly lifestyle among our guilds of cetaceans. The most abundant genera (*Stenella spp* and *Tursiops spp*) are known to largely forage at night, mainly from the surface to 200 m, following both vertical and horizontal migration of mesopelagic micronekton [55]–[57]. These two genera also perform regular or more occasional excursions down to 400–500 m [56], [58]. Given these characteristics, we expected *Delphininae* to forage on epi- and mesopelagic prey (Figure 1A).

**Figure 8:**
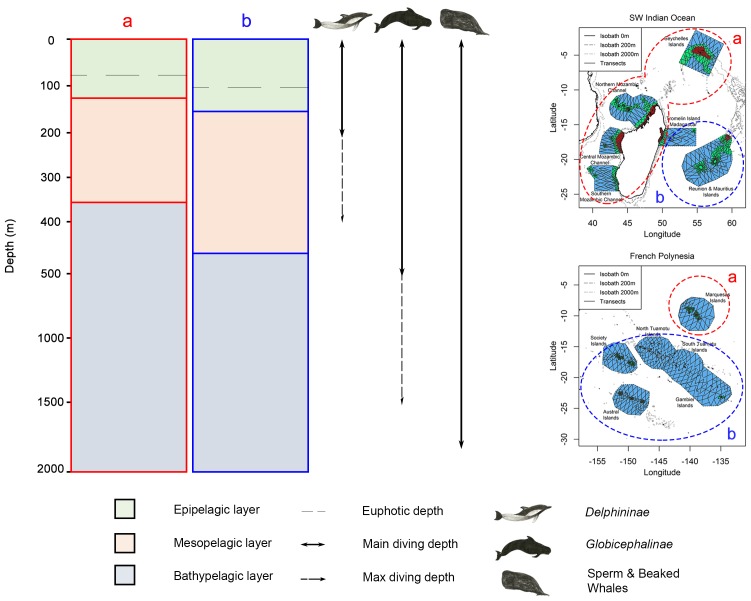
Water column structure in productive (a) and oligotrophic (b) areas of the South West Indian Ocean and French Polynesia. The diving abilities of the three cetacean guilds (*Delphininae*, *Globicephalinae* and sperm and beaked whales) are indicated. Corresponding productive and oligotrophic areas are delimited in the two study regions on the right panel.

In the SWIO, *Delphininae* showed an heterogeneous predicted distribution (a ratio of 17.8 between sectors), with the highest relative predicted densities in productive waters. As euphotic depth was the most contributory covariate, *Delphininae* appeared to select areas primarily according to prey accessibility. The shallowest euphotic depth in the SWIO was 80 m, consequently the boundary between epi- and mesopelagic layers was 120 m, resulting in an easily accessible mesopelagic layer for *Delphininae* (Figure 8). However, shallow euphotic depth areas were concomitant with a high mesopelagic biomass, and deep euphotic depth areas with a low mesopelagic biomass. The mesopelagic biomass was the second most contributory covariate with a positive relationship, hence our results indicates a clear preference for areas where *Delphininae* might optimise their foraging success *i.e.* where mesopelagic biomass was the highest and the most accessible.

In FP, euphotic depth was less contributory than in the SWIO, which may be an indication that *Delphininae* may have selected foraging areas more on the basis of prey density than accessibility. In this oligotrophic region, epi- and mesopelagic layers were poorer than in the SWIO. To sustain their foraging success *Delphininae* might have to forage in the bathypelagic layer, where overall prey biomass/production were higher. However, the bathypelagic layer was deeper than the average foraging depth of *Delphininae* (200 m, Figure 8), thus they might not sustain the costs induced by foraging at such a depth on a regular basis. Therefore, the overall foraging conditions in FP were not in favour of the guild, explaining why the overall relative density in FP was on order of magnitude lower than in the SWIO.

*Globicephalinae* exhibited similar spatial patterns to *Delphininae*, but appeared to select areas on a different basis. In the SWIO, high densities of *Globicephalinae* were found in areas with intermediate epipelagic production and shallow euphotic depth. Similar to *Delphininae*, *Globicephalinae* appeared to select areas where high production was the most accessible. High densities were predicted on the slope of the Seychelles and southern Madagascar, in accordance with previous field observations [59], [60].

In FP, the bathypelagic biomass was the most contributory predictor, with a similar relationship as for *Delphininae*, whereas the other three covariates had low contributions and little effects on density. *Globicephalinae* might select this deep layer for the same reason as *Delphininae*. However, in contrast to *Delphininae*, this layer was probably more accessible for *Globicephalinae* which can dive deeper (Figure 8): up to 1,500 m for *Peponocephala electra* [61], the most frequently identified species both in the SWIO and in FP, 1,019 m at dawn and dusk for *Globicephala macrorhynchus* [62] and 700 m for *Pseudorca crassidens* [63]. Due to their greater diving abilities, the bathypelagic layer would have been more accessible to *Globicephalinae* even in deep euphotic depth areas (Figure 8). This could explain the more homogeneous predicted distribution compared to *Delphininae*, as *Globicephalinae* could forage in the bathypelagic layer even in southern FP.

### LER cetaceans (sperm and beaked whales)

Distribution patterns differed for sperm and beaked whales which have the lowest cost of living. In the SWIO, they showed a slight preference for areas with a high production. However, the low ratio (2.1) of the mean relative predicted densities between sectors may be an indication that sperm and beaked are less dependent with respect to variations in prey availability, and suggest that they could sustain their needs in both productive and oligotrophic areas. In FP, this ratio was higher (6.1), because relative densities were higher around the Marquesas compared to the other sectors. However, in FP, the mean relative predicted densities were the same as those in the SWIO: about 5.10^−4^.

Beaked whales forage between 460 and 1,890 m [64], [65], while sperm whales forage between 400 and 1,300 m [66], [67] and *Kogidae* potentially forage up to 1,500 m, as inferred from their diets [68] (Figure 8). Since these foraging depth ranges encompass the three vertical layers defined in SEAPODYM, our results agree with the observed behaviours of these deep-diving species. In both regions, the most contributory covariates of habitat models corresponded to deep layers, *i.e.*, either mesopelagic (SWIO) or bathypelagic (FP), with positive relationships. The relationships for the other selected variables were negative and thus more difficult to explain. A negative relationship with the distribution of epipelagic production might reflect a general preference for clear and deep waters rather than shallow coastal waters. Finally, in agreement with their low costs of living, the relative predicted densities of sperm and beaked whales showed low variations both within and between regions.

### Comparison with previous studies

Very few dedicated surveys have been conducted in the SWIO and FP to document cetacean abundance and distribution [69]. Mannocci and colleagues [11], [12] conducted the first habitat modelling studies at the scale of these regions, relying on the same sightings, hypotheses and modelling procedure as presented here, but using oceanographic and static predictors, whereas we used simulated micronekton distributions as predictors.

Direct comparison between the two modelling approaches is difficult, as modelling by Mannocci and colleagues [11], [12] was based on a smaller sampling unit (the 10 km-transect segment as compared to the 0.25°×0.25° pixel in the present study). Despite this difference, the two studies yielded fairly similar prediction maps [11], [12]. In particular, predictions for *Delphininae* show the highest densities in the Mozambique Channel and the Seychelles in the SWIO, and around the Marquesas in FP. Both studies predicted very low densities of sperm and beaked whales in the SWIO and FP compared to the other two guilds, with the highest densities on the slope in the SWIO, and around the Marquesas in FP. Results were more divergent for *Globicephalinae*: Mannocci and colleagues [11] predicted intermediate densities to the east of Madagascar, whereas our models predicted low densities in this area.

To date, SEAPODYM has only been used to predict tuna populations dynamics [13], [16]–[18], albacore tuna catch rates [14] and the habitat preferences of sea turtles [15]. Our study is the first to use the simulated distribution of micronekton as a predictor of cetacean habitats. Such predictors provide additional insights into cetacean distribution, by integrating the food web, as micronekton is at least two trophic levels closer to cetaceans than primary production. In addition, as depth is a key factor in cetacean foraging strategies, considering the vertical dimension in the models by using micronekton functional groups appears to improve cetacean habitat models.

The relationships of marine predators with their prey have already been studied at the fine scale, notably at the prey patch level. Torres and colleagues [70] demonstrated that habitat modelling of dolphins was more successful with environmental variables than with prey distributions. This was explained by habitat heterogeneity at the fine scale, coincident with the patchiness of prey distribution. Benoit-Bird and colleagues [71] also studied the spatial relationships between predators and their prey for two seabird and one seal species. They demonstrated the importance of prey patch characteristics, but found no direct spatial relationship between predators and their prey. These studies indicated that, at small scale, prey patch characteristics (the physical habitat inducing prey aggregation) appears to be more important for predators than the overall prey biomass. However, processes highlighted at such small spatial scales refer to foraging behaviour and are not readily accessible in large-scale sighting surveys.

At a broader scale, Hazen and Johnston [72] studied the relationships between cetacean sightings and oceanographic and biological variables collected along a transect in the central Pacific. They used acoustic echo-sounding to study the spatial distribution of the deep scattering layer. Cetaceans were related to the density of the deep scattering layer with respect to their diving capacities: pilot whales distribution was correlated with high acoustic densities of prey in the mid- and deep layers, whereas *Stenella* dolphins and false killer whales were associated with a shallower backscattering layer. Hazen and colleagues [73] developed a habitat model of *Mesoplodon spp* in the Bahamas, based on the detection of foraging clicks. The resulting model (Generalised Linear Model) explained 54% of the variance, with five significant covariates: bottom depth, salinity, temperature, prey density and number of single targets. The latter two covariates had a positive effect on foraging activity, and their depth matched the known foraging depth of beaked whales. Although these two studies are based on *in situ* prey data, their conclusions are similar to ours, which, in contrast, rely on simulated prey distributions: cetaceans directly respond to the horizontal and vertical distributions of their prey in accordance with their known diving abilities.

### Modelling considerations

GAMs are common tools in habitat modelling, given their ability to model non-linear relationships [2]. Our models are based on large sample sizes (1,473 pixels were used for modelling in the SWIO and 1,821 in FP), with a large number of presences (see [16], [17] and Figure S1), thus providing a good statistical basis. Nevertheless, the ecological interpretation of complex relationships detected through GAMs can be difficult and need to be analysed with caution.

Low explained deviances are common in GAMs, especially for cetaceans. For example, in the California Current Ecosystem, Becker and colleagues [74] obtained deviances ranging from 5 to 32% for delphinids and a deviance of 5% for the sperm whale with their encounter rates models, with six oceanographic potential variables in the model selection procedure and three degrees of freedom. In the Eastern Tropical Pacific, Forney and colleagues [75] obtained deviances ranging from 5 to 8% for sperm and beaked whale and from 8 to 28% for delphinids species with their encounter rates models, with nine oceanographic potential variables and three degrees of freedom. Deviances of the models built by Mannocci and colleagues [11], [12] ranged between 9.6 and 30% for *Delphininae* and *Globicephalinae* and were about 5% for sperm and beaked whales. In comparison, our models resulted in higher explained deviances, ranging from 18 to 47% for *Delphininae* and *Globicephalinae*, and from 9 to 13% for sperm and beaked whales. This suggests that incorporating prey data might improve cetacean habitat models.

It should be kept in mind that the prey data used here are model outputs, and thus include a degree of uncertainty. Even if the ocean reanalysis was produced with data assimilation techniques, the physical forcing (especially the currents) is not always fully consistent with *in situ* measurements, particularly in the deeper layer and the equatorial region (*e.g.*, [76]). The VGPM model used to compute vertically integrated primary production provides an estimate inferred from empirical relationships based on surface chlorophyll content [77]. The parameterisation of coefficients of energy transfer from primary production to functional groups is still preliminary and under revision using parameter optimisation approach with large datasets of acoustic data.

Nevertheless, the similar predicted distributions obtained when using SEAPODYM outputs and static and oceanographic variables [11], [12], suggest that SEAPODYM micronekton outputs may be accurate enough to provide robust predictions at the regional scale. However, it should be noted that in areas or depths where micronekton functional groups are not modelled by SEAPODYM (*e.g.*, bathypelagic functional groups over the continental shelf, where by definition the deepest layer does not exist), we were not able to provide predictions of cetacean densities. This might be important for *Delphininae*, given that this guild contains several species with extensive coastal populations [78], [79]. This limitation does not concern studies based on static and oceanographic covariates, such as Mannocci and colleagues' [11], [12].

### Conclusion

This study confirms the findings of Mannocci and colleagues [11], [12] concerning the habitat selection of cetaceans. *Delphininae* and *Globicephalinae*, with relatively high costs of living, showed a strong dependency on prey biomass and production. In the productive but contrasting SWIO, they might optimise their foraging strategies by selecting areas where biomass and production are the highest and the most accessible. In the oligotrophic FP, they might forage in the deeper layers to sustain their energy requirements, which might induce greater foraging costs and explain their lower densities. The deep layer accessibility might even become a limiting factor for *Delphininae* occurrence in regions of lower productivity. Conversely, for sperm and beaked whales with the lowest costs of living, we did not find any strong differences within and between regions, indicating that they might be able to sustain their energy requirements by exploiting both productive and oligotrophic areas. This apparent lack of preference for productive areas might be due to their deep diving abilities, emancipating them from the constraint induced by the strong variability in resources in upper layers, whereas in the bathypelagic layer micronekton is more evenly distributed across the ocean basins.

The use of prey biomass and production as predictors in cetacean habitat models provided encouraging results. The relationships between cetaceans and their potential prey are consistent with the results of acoustic-based studies, which relate cetacean occurrence to estimated *in situ* prey densities [72], [73]. Our study demonstrates that simulated micronekton distributions are valuable for understanding cetacean strategies of habitat utilisation in both the horizontal and the vertical dimensions, and therefore are useful for predicting cetacean habitats without conducting expensive surveys of prey distributions.

## Supporting Information

Figure S1 **Numbers of individuals observed per sampled pixel for each cetacean guild in the two study regions.** Sightings observed in the South West Indian Ocean are presented in panel A, sightings in French Polynesia in panel B. The 25th, 50th, 75th quartiles and the maximum number of individuals per pixel are indicated at the top left of each map. For details on the sightings, see [11], [12].
(TIF)

Figure S2 **Euphotic depth (m) in the South West Indian Ocean (A) and French Polynesia (B).** Values were averaged over the survey period of each region.
(TIF)

Figure S3 **SEAPODYM micronekton outputs in the South West Indian Ocean.** Values were averaged over the survey period for the whole region. Covariates were log-transformed before modelling.
(TIFF)

Figure S4 **SEAPODYM micronekton outputs in French Polynesia.** Values were averaged over the survey period for the whole region. Covariates were log-transformed before modelling.
(TIFF)

Table S1 **Surveyed effort (km) and observed encounter rates (individuals per km^2^) of cetacean guilds in each sector in the two study regions.**
(PDF)

Table S2 **Correlation matrix for SEAPODYM covariates in the South West Indian Ocean (in red) and French Polynesia (in blue).** Correlations were calculated with Hmisc package using the Spearman correlation test in R.
(PDF)

Text S1 **Micronekton outputs description**.
(PDF)
