# Supplementary figures and images for: Predicting Cetacean Habitats from Their Energetic Needs and the Distribution of Their Prey in Two Contrasted Tropical Regions
Source: PLoS One. 2014 Aug 27;9(8):e105958. doi: 10.1371/journal.pone.0105958 (PMC4146581; doi:10.1371/journal.pone.0105958)

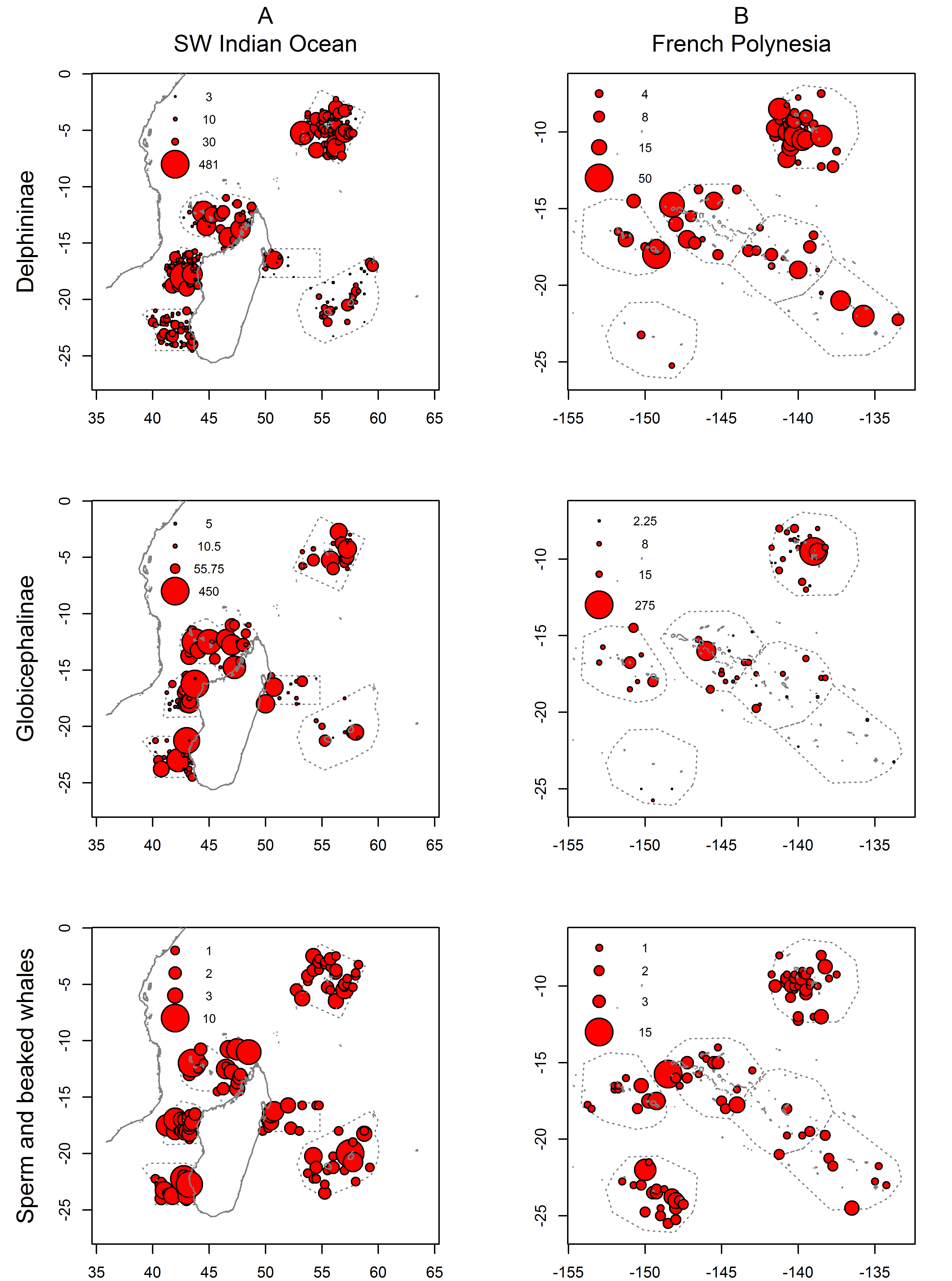

Supplement: Figure S1 — Numbers of individuals observed per sampled pixel for each cetacean guild in the two study regions. Sightings observed in the South West Indian Ocean are presented in panel A, sightings in French Polynesia in panel B. The 25th, 50th, 75th quartiles and the maximum number of individuals per pixel are indicated at the top left of each map. For details on the sightings, see [11], [12]. (TIF) [file pone.0105958.s001.tif]

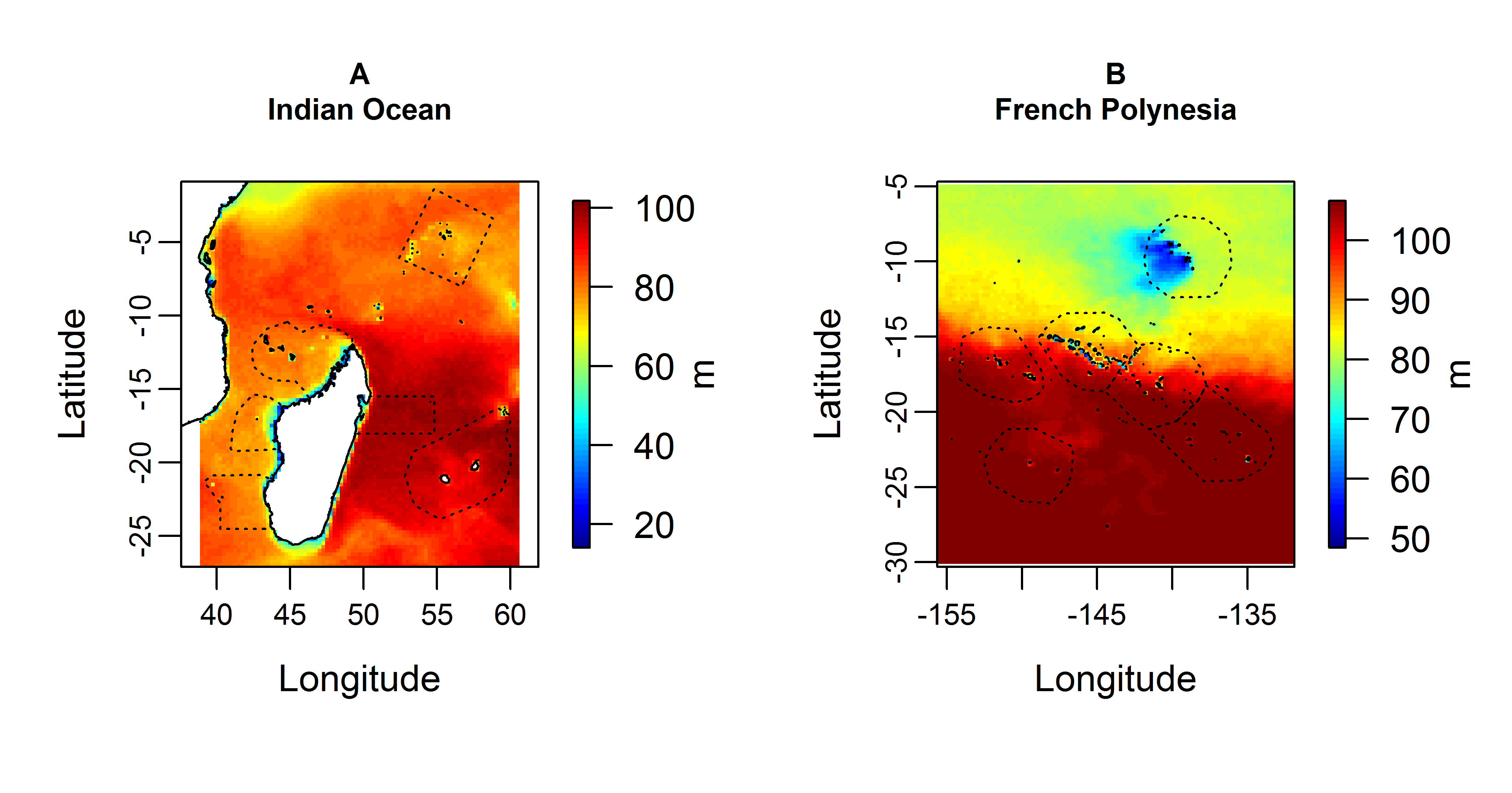

Supplement: Figure S2 — Euphotic depth (m) in the South West Indian Ocean (A) and French Polynesia (B). Values were averaged over the survey period of each region. (TIF) [file pone.0105958.s002.tif]

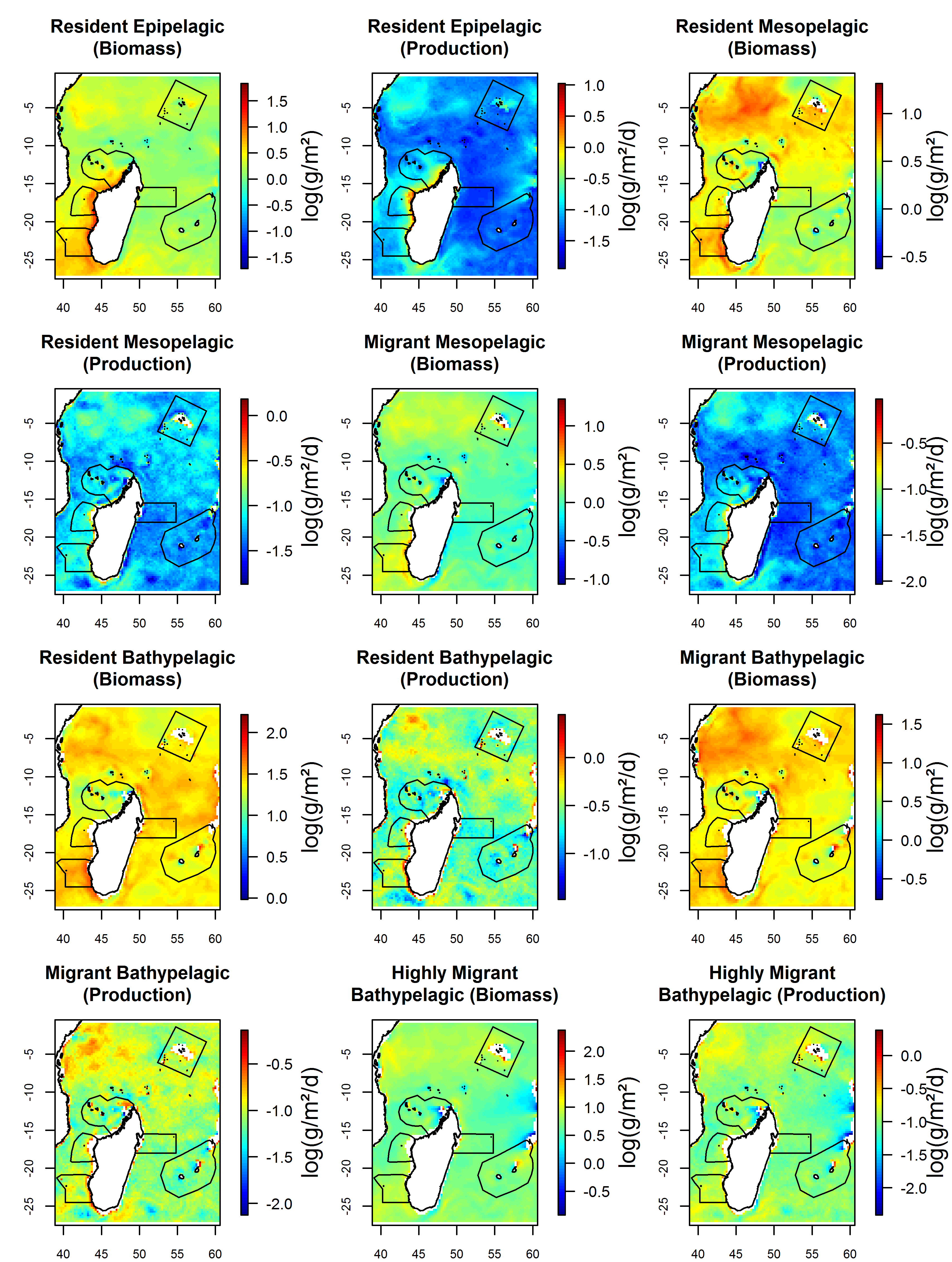

Supplement: Figure S3 — SEAPODYM micronekton outputs in the South West Indian Ocean. Values were averaged over the survey period for the whole region. Covariates were log-transformed before modelling. (TIFF) [file pone.0105958.s003.tiff]

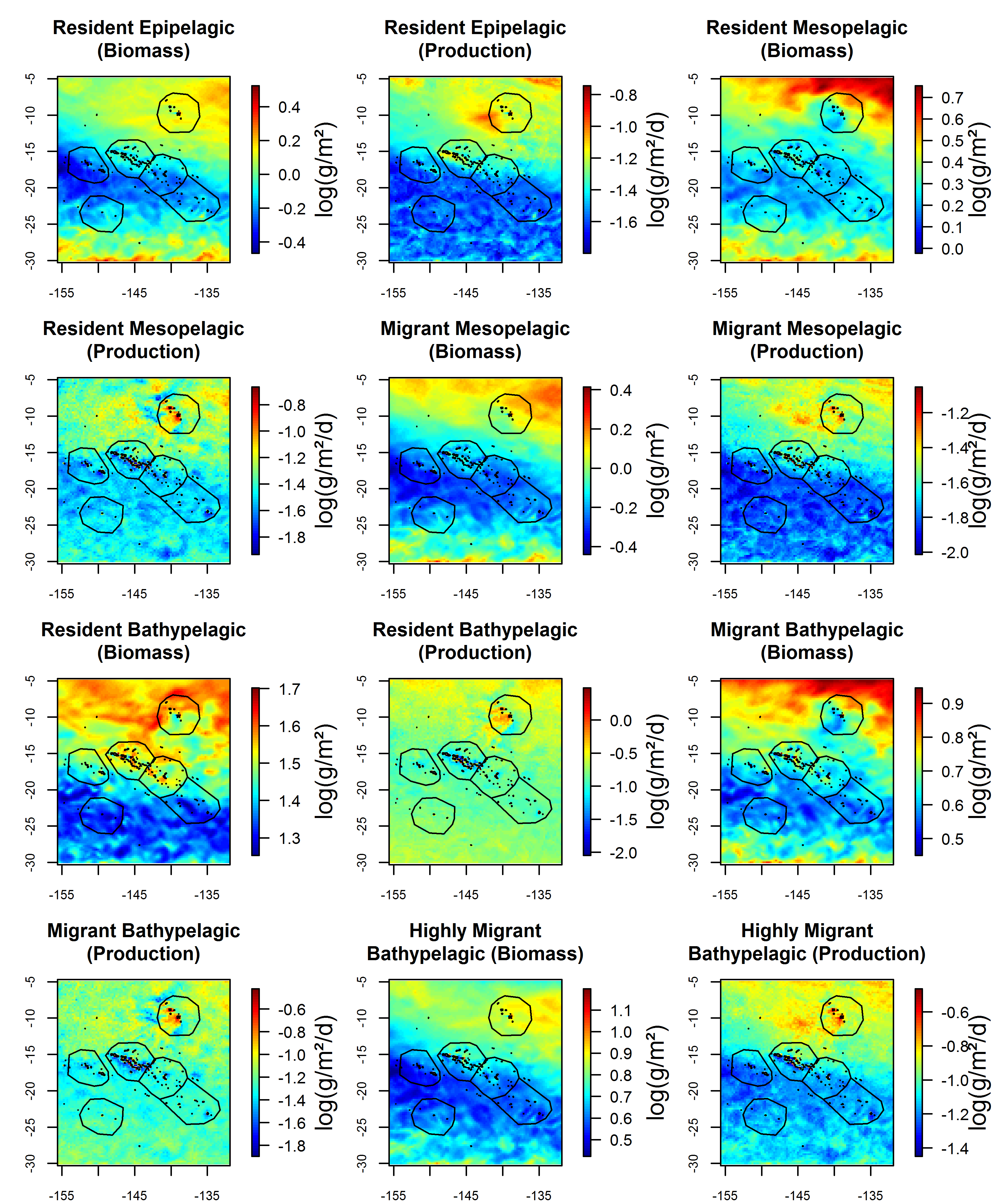

Supplement: Figure S4 — SEAPODYM micronekton outputs in French Polynesia. Values were averaged over the survey period for the whole region. Covariates were log-transformed before modelling. (TIFF) [file pone.0105958.s004.tiff]
