# Supplementary material for: Predicting Cetacean Habitats from Their Energetic Needs and the Distribution of Their Prey in Two Contrasted Tropical Regions
Source: PLoS One. 2014 Aug 27;9(8):e105958. doi: 10.1371/journal.pone.0105958 (PMC4146581; doi:10.1371/journal.pone.0105958)
Supplement: Table S1 — Surveyed effort (km) and observed encounter rates (individuals per km2) of cetacean guilds in each sector in the two study regions. (PDF) [file pone.0105958.s005.pdf]

Table S1. Surveyed effort (km) and observed encounter rates (individuals per km²) of cetacean guilds in each sector in the two study regions.

South West Indian Ocean					French Polynesia				
Sectors	Effort (km)	Encounter rates (individuals per km²)			Sectors	Effort (km)	Encounter rates (individuals per km²)		
		Delphininae	*Globicephalinae*	Sperm and beaked whales			*Delphininae*	*Globicephalinae*	Sperm and beaked whales
CMGM	15198	0.292	0.301	0.009	MAR	16876	0.039	0.049	0.008
EBM	9785	0.243	0.202	0.009	TUN	15385	0.039	0.049	0.008
JNM	9776	0.612	0.224	0.007	TUS	13815	0.009	0.013	0.002
RM	24087	0.040	0.012	0.003	GAM	13285	0.011	0.002	0.002
SE	14448	0.463	0.226	0.006	SOC	16390	0.009	0.017	0.002
TM	10432	0.030	0.077	0.007	AUS	22726	0.0003	0.0004	0.003
Total	*83726*	*0.282*	*0.176*	*0.007*	*Total*	*98477*	*0.018*	*0.022*	*0.004*
