# Supplementary material for: Predicting Cetacean Habitats from Their Energetic Needs and the Distribution of Their Prey in Two Contrasted Tropical Regions
Source: PLoS One. 2014 Aug 27;9(8):e105958. doi: 10.1371/journal.pone.0105958 (PMC4146581; doi:10.1371/journal.pone.0105958)
Supplement: Table S2 — Correlation matrix for SEAPODYM covariates in the South West Indian Ocean (in red) and French Polynesia (in blue). Correlations were calculated with Hmisc package using the Spearman correlation test in R. (PDF) [file pone.0105958.s006.pdf]

Table S2. Correlation matrix for SEAPODYM covariates in the South West Indian Ocean (in red) and French Polynesia (in blue). Correlations were calculated with Hmisc package using the Spearman correlation test in R.

		Biomass						Production						Euphotic depth
		Bathypelagic	Epipelagic	Highly migrant bathypelagic	Migrant bathypelagic	Mesopelagic	Migrant mesopelagic	Bathypelagic	Epipelagic	Highly migrant bathypelagic	Migrant bathypelagic	Mesopelagic	Migrant mesopelagic	
Biomass	Bathypelagic	1	0.400	0.609	**0.804**	0.271	0.365	0.094	0.715	0.412	0.170	0.325	0.623	-0.737
	Epipelagic	0.254	1	**0.882**	0.406	0.566	**0.925**	0.146	0.750	0.697	0.187	0.404	0.701	-0.519
	Highly migrant bathypelagic	0.410	0.336	1	0.634	0.615	**0.890**	0.181	**0.801**	0.744	0.266	0.459	0.760	-0.625
	Migrant bathypelagic	**0.772**	0.392	0.590	1	0.647	0.487	0.154	0.593	0.434	0.230	0.374	0.541	-0.542
	Mesopelagic	0.434	0.571	0.434	**0.711**	1	0.764	0.317	0.412	0.511	0.326	0.451	0.428	-0.260
	Migrant mesopelagic	0.338	0.687	0.645	0.583	**0.820**	1	0.234	0.678	0.721	0.272	0.478	0.677	-0.466
Production	Bathypelagic	0.268	0.115	0.291	0.190	0.099	0.150	1	0.061	0.410	0.768	0.566	0.249	-0.174
	Epipelagic	-0.005	0.642	0.188	0.117	0.272	0.404	0.207	1	0.609	0.171	0.406	0.784	-0.787
	Highly migrant bathypelagic	0.195	0.266	0.566	0.344	0.290	0.449	0.460	0.336	1	0.476	0.677	0.727	-0.594
	Migrant bathypelagic	0.201	0.209	0.254	0.296	0.267	0.262	0.665	0.349	0.695	1	0.713	0.379	-0.229
	Mesopelagic	0.100	0.351	0.211	0.267	0.405	0.390	0.483	0.463	0.615	**0.826**	1	0.652	-0.510
	Migrant mesopelagic	0.135	0.516	0.438	0.289	0.434	0.620	0.414	0.624	0.652	0.611	**0.734**	1	-0.746
Euphotic depth		-0.017	-0.527	-0.163	-0.155	-0.308	-0.425	-0.332	-0.521	-0.197	-0.202	-0.312	-0.429	1
