# Supplementary material for: Predicting Cetacean Habitats from Their Energetic Needs and the Distribution of Their Prey in Two Contrasted Tropical Regions
Source: PLoS One. 2014 Aug 27;9(8):e105958. doi: 10.1371/journal.pone.0105958 (PMC4146581; doi:10.1371/journal.pone.0105958)
Supplement: Text S1 — Micronekton outputs description. (PDF) [file pone.0105958.s007.pdf]

Text S1. **Micronekton outputs description**.

Micronekton distributions provided by SEAPODYM were composed of 12 functional groups for each region. Predicted biomass and production were the highest in the deepest layer groups (non-migrant, migrant and highly migrant bathypelagic groups) compared to the 3 others groups in all regions, except in the SWIO, where the epipelagic production showed higher maximum values than non-migrant bathypelagic production.

In the SWIO, the distributions of epipelagic and mesopelagic functional groups were quite contrasting, with high biomass and production in the Mozambique Channel, off Kenya and around the Seychelles, as opposed to the less variable bathypelagic micronekton groups (Supporting Information, Figure S3). In FP, all functional groups reflected the latitudinal gradient characterising the region (Supporting Information, Figure S4). Higher biomass and production were found for all micronekton groups in the north of the region, although the production of the non-migrant bathypelagic and migrant bathypelagic functional groups was less variable than for the others groups. Epipelagic, non-migrant mesopelagic and migrant mesopelagic functional groups were also characterised by fairly high biomass at the extreme south of FP.
